# Pathology of HPV-Associated Head and Neck Carcinomas: Recent Data and Perspectives for the Development of Specific Tumor Markers

**DOI:** 10.3389/fonc.2020.528957

**Published:** 2020-11-16

**Authors:** Xavier Sastre-Garau, Alexandre Harlé

**Affiliations:** ^1^ Service de Pathologie, Centre Hospitalier Intercommunal de Créteil, Créteil, France; ^2^ Université de Lorraine, CNRS UMR7039 CRAN, service de Biopathologie, Institut de Cancérologie de Lorraine, Vandoeuvre-Lès-Nancy, France

**Keywords:** HPV—human papillomavirus, head and neck carcinoma, ctDNA, tumor markers, tumor microenvironment, viral integration, viral oncogenesis

## Abstract

A significant subset of carcinomas developed in the head and neck (H&NCs) are associated with specific human papillomaviruses (HPV) genotypes. In particular, 40–60% of oropharyngeal carcinoma cases are linked to HPV. Epidemiological studies have demonstrated that HPV oral infections are predominantly sexually transmitted and are more frequent among men (10–18%) than women (3.6–8.8%). Although there is a large diversity of HPV genotypes associated with H&NCs, HPV16 lineage represents 83% of the reported cases. The prognostic value of HPV as a biological parameter is well recognized. However, the use of HPV DNA as a diagnostic and/or predictive marker is not fully developed. Recent data reporting the physical state of the HPV genome in tumors have shown that HPV DNA integration into the tumor cell genome could lead to the alteration of cellular genes implicated in oncogenesis. Most importantly, HPV DNA corresponds to a tumor marker that can be detected in the blood of patients. Profile of the HPV DNA molecular patterns in tumor cells using New Genome Sequencing-based technologies, allows the identification of highly specific tumor markers valuable for the development of innovative diagnostic and therapeutic approaches. This review will summarize recent epidemiological data concerning HPV-associated H&NCs, the genomic characterization of these tumors, including the presence of HPV DNA in tumor cells, and will propose perspectives for developing improved care of patients with HPV-associated H&NCs, based on the use of viral sequences as personalized tumor markers and, over the longer term, as a therapeutic target.

## Introduction

The first aim of this review is to summarize recent data concerning the pathology of head and neck carcinomas (H&NCs) associated with the human papillomaviruses (HPV), including prevalence and viral epidemiology which characterize these tumors as well as the specificities of HPV DNA as a tumor marker. In the second aim, we will explore specific perspectives focused on the use of HPV DNA as a prognostic tumor marker in the blood of patients with H&NCs and on the developments of new applications in clinical oncology related to the introduction of New Genome Sequencing (NGS) approaches as tools for the optimized characterization of viral DNA in the tumor cells of HPV-positive H&NCs.

## Epidemiology

### Epidemiology of HPV-Associated H&NCs

The reported worldwide prevalence of HPV-associated H&NCs varies between 25.9 (1) and 30% (2). The frequency of HPV association is different according to tumor localizations. The highest rate (35%) is observed for tumors located in the oropharynx (1, 3), particularly when developed in lympho-epithelial sites such as palatine tonsil (56 to 62%) (4, 5) and base of the tongue (40%) (4). Lower rates are observed in tumors developed in the oral cavity, (5.8 to 23.5%) (1, 3), in the larynx (3.3 to 24.0%) (1, 3), or in the soft palate (3.1%) (6).There is a striking geographic heterogeneity of HPV prevalence in oropharyngeal tumors: rates are higher in the United States (59.3%) and in Europe (31.1%) than in Brazil (4.1%) (3, 7). In the United States, an increase in the prevalence of H&NCs has been observed between 1984–1989 and 2000–2004, rising from 16.3 to 71.7% (8). A significant increase of HPV-related tumors developed in women has also been observed in France (9).

### Viral Epidemiology of Asymptomatic Oral Infection and of H&NCs

In the general population, the prevalence of asymptomatic HPV infection was assessed by viral analyses of oral rinse specimens. The reported infection statistics ranged from 6.9% in the United States (10) to 13.1% in France (11). The prevalence is two- to three-fold higher in men (from 10.1 to 18%) (10–12) than in women (3.6 to 8.8%) (10, 11). Within non-tumor tonsils, HPV DNA was found in 3.6 to 4.9% from these specimens (11, 13). Paired analyses of tonsil brushing and oral rinse found a low agreement between the results (11) indicating that oral rinse, not able to reach the bottom of all of the tonsillar crypts, was a poor surrogate of HPV prevalence within tonsils. In a Japanese study concerning male patients, HPV DNA detected from mouth rinses was also found in the urine with a good agreement between the genotypes detected in these two specimens from the same patient (12).

The mode of contamination has been further analyzed from a large cohort study conducted with 5,500 subjects in the United States (10). This study showed that oral HPV infection was predominantly sexually transmitted: oral HPV prevalence was more than 8-fold higher among individuals who reported sexual relations *vs* no sexual relations (7.5 *vs* 0.9%). The prevalence increased with the number of partners but no evidence for increased risk related to particular sexual behavior was observed. However, one explanation for the observed higher prevalence among men could be attributed to the higher probability of HPV transmission through oral sex with women *vs* men. Indeed, oral HPV prevalence increased more sharply with the number of sexual partners for men than women (10). Low-risk HPV genotypes were two-fold more frequently detected than high-risk. Multivariate analyses showed that HPV prevalence was also related to cigarette smoking and to age, with a bimodal pattern characterized by peak prevalences among individuals aged between 30 to 34 and 60 to 64 years (10).

Patients with oropharyngeal cancer showed risk factors including a history of numerous sexual partners and oral sex (14–16). A study analyzing the risk of cancer among homosexual individuals showed an increased prevalence of oropharyngeal cancer in women but not in men (17). No significant increase in HPV infection was observed in partners of H&NCs patients (18).

When looking at H&NCs as well as cervical cancers, there is a large heterogeneity of the high-risk HPV genotypes encountered, but the prevalence of HPV16 in H&NCs (83.0%) (19) is significantly higher than in the cervix (55.5%) (20). In H&NCs, the other high-risk genotypes are found at lower rates: 3.3% for HPV33, 2.6% for HPV26, 2.2% for HPV35, 1.8% for HPV18, and below 1% for the other genotypes. In laryngeal tumors, the distribution of the respective genotypes is 50.8% for HPV16, 8.5% for HPV45, 6.6% for HPV6, 5.1% for HPV18, 3.4% each for HPV31 and HPV33, 1.7% for HPV35, and less than 1% for the other genotypes (19).

The improvement in sequencing technologies has revealed a high diversity of HPV16 DNA sequences. Four major variant lineages and up to 16 sublineages were identified (21), associated with different risks of persistence and of progression to invasive carcinoma (22) and with distinct histological types (23). Recently, among a series of 5,570 HPV16-infected case-control cervical samples, Mirabello et al.** identified thousands of unique HPV16 viral isolates (24). In contrast to this variability, the HPV16 E7 gene showed extremely low variability in cervical cancers around the world, indicating that genetic conservation of this viral oncogene is critical for carcinogenesis. The role of HPV genome variants in the development of H&NCs merits updated and precise documentation in the HPV community (11).

## Genetics of HPV Positive and HPV Negative H&NCs

Independently of their HPV status, H&NCs are characterized by recurrent mutations in the *TP53, CDKN2A, PI3KCA, HRAS, NOTCH1*, and *FBXW7* genes (25, 26) and in at least 30% of the cases, harbor mutations in genes regulating squamous cell differentiation, such as *NOTCH1, IRF6*, and *TP63* (27). Recurrent overexpression of sequences corresponding to relevant therapeutic targets, such as *PGF, PDL1, CDK6, MET*, and *EGFR*, were also observed (28).

Several studies aimed at determining the genetic alterations that distinguish HPV-positive from HPV-negative H&NCs (26, 27, 29, 30). In HPV-positive tumors, the mutation rate was two-fold lower (2.28 mutations per Mb) than in HPV-negative tumors (4.83 per Mb) (27). HPV-positive tumors harbor recurrent mutations of *PTEN*, *TRAF3* (TNF receptor associated factor 3), and *PIK3CA* and focal amplification of *E2F1* whereas HPV-negative cases are characterized by a high rate of *TP53* mutations and abrogation of the G1/S checkpoint *via CDKN2A/B* deletion and/or *CCND1* amplification. In line with an enhancement of cell proliferation induced by the E7 HPV protein (31), genome-wide expression profile of HPV-positive H&NCs revealed an up-regulation of a distinct and large subset of cell cycle genes as compared with HPV-negatives cases (32). In summary, these analyses show that HPV-positive H&NCs are mainly characterized by a low mutational load, a high proliferative index, integrity of p53, and a frequent alteration of the *PTEN/PIK3CA* pathway.

The HPV genome is a 7.8-kbp double-stranded DNA circular molecule and its presence in the nucleus of tumor cells represents *per se* a genetic alteration. In cervical neoplasias, the analyses of the interactions between viral and cell genomes have shown that the physical state of HPV genomes in tumor cells is different according to the types of lesions. In benign or in intraepithelial lesions, viral genomes are present as free episomal molecules in the nucleus of infected cells whereas, in most invasive cancers, part of the viral DNA is integrated into the cell genome (33). HPV DNA integration is clonal, stable over time and, homogeneously distributed throughout different tumor regions, does not depend upon intra-tumor heterogeneity (34). Host chromosomal structural alterations at the HPV integration locus are frequently observed (35) and the nature of these changes is related to the recombination mechanisms of viral insertions (36). These chromosomal alterations, as well as the introduction of illegitimate viral DNA enhancer sequences into the cell genome, have consequences on the expression of cellular genes located near integrated viral sequences (37). Tumor cells may also contain non-integrated episomal molecules in various quantities, ranging from a few to several thousand per nucleus (38).

For H&NCs, few analyses have been performed concerning the physical state of HPV genomes, but the reported pattern is similar to that observed in genital carcinomas. Viral integration was detected in 60.7% (51/84) (39) to 71.4% (25/35) (40) of the cases, although a low prevalence of 15.4% (2/13) was observed in another series (41). As in genital tumors, integration was frequently found within or in close vicinity (<20 Kb) to cellular genes and tumors without viral integrants displayed distinct gene expression profile (40). Reported target genes for HPV insertion were *RAD51B*, *ETS2*, *NR4A2*, *KLF5*, *KLF12*, *p63*, *CD274*, *FLJ3745*, and *TTC6* (39, 40).

## HPV as a Tumor Marker in H&NCs

### Prognostic and Predictive Value of HPV DNA in H&NCs

Many strategies for HPV characterization in H&NCs have been used (42, 43) including Polymerase Chain Reaction (PCR) or Quantitative Reverse Transcription-PCR (qRT-PCR) for the direct detection of HPV DNA or RNA, and immunohistochemistry (IHC) for the detection of P16INK4A (p16) cell protein. Viral E7 oncoprotein expressed from high-risk HPVs inactivates cellular Rb protein leading to the up-regulation of various cell cycle associated protein, including p16, which is commonly used as an indirect surrogate marker of HPV infection. Consistent discrepancies in the respective positivity of p16 and HPV DNA/RNA have been reported (44). Due to the high sensitivity and specificity of PCR and qRT-PCR, these methods are widely accepted for the clinically significant detection of HPV infection in H&NCs but p16 IHC is commonly used as a complementary procedure due to its low cost and sensitivity (43). Guidelines suggest to perform p16 IHC as the initial test for HPV characterization in tumor tissue, followed by additional molecular test at the discretion of pathologists (45). It was shown that combined p16 and HPV DNA testing discriminates subgroup of tumors with significantly distinct outcome (44, 46).

The outcome of HPV-associated H&NCs is better than that of HPV-negative cases (47). In a meta-analysis including 42 studies, both progression-free survival and disease-free survival were significantly improved in HPV-positive tumors (48). However, the prognostic value of the HPV status should be appreciated differently according to tumor histology. Most HPV-positive H&NCs correspond to SCC, but undifferentiated carcinomas may also be HPV-associated and present a favorable outcome (49) whereas large cell neuro-endocrine carcinoma (50) and high-grade neuro-endocrine carcinomas (51) correspond to diseases of poor outcome whatever their HPV status may be. Neuro-endocrine tumors frequently express p16 and this marker is a poor surrogate for HPV-association in these tumors (52). In addition, H&NCs associated with HPV genotypes other than HPV16 were found to have unfavorable outcomes as compared with HPV16-positive cases (53), an inversed association to that observed in cervical cancers (20). Integration pattern might also be clinically predictive: H&N squamous cell carcinoma with presence of episomal form of the viral genome without integration were associated with a better outcome than HPV integration-positive or HPV-negative cases (39) and tumor relapse was more frequently observed when HPV-DNA was found inserted in cancer-related genes rather than in intergenic loci (54).

In patients with carcinomas of the oral cavity or of the oropharynx, the oral rinse sampling for prognostic purpose may represent a convenient approach for sequential viral analyses. A diagnostic rate of 81 and 100% of sensitivity and specificity for HPV-16-positive cases was observed (55). After completion of primary therapies, the presence of persisting viral DNA in the oral cavity was associated with an increased risk of recurrence: the disease-free survival at 2 years was 55% in patients with persistent HPV detection *versus* 88% for viral-negative cases (55).

It is likely that the HPV status has also a predictive value. A specific HPV-related tumor immune microenvironment (56) may be implicated in improving sensitivity to treatments. The analysis of the immune infiltrate in H&NCs has shown that HPV-positive cases exhibited greater CD8+ T cell infiltrate and PD-L1 expression than HPV-negative tumors (57–60). This immune pattern was associated with favorable outcome after chemo-radiotherapy (58). Moreover, clinical trials provide data indicating that anti-PD-1/PD-L1 therapy results in anti-tumor activity in H&NCs (61) and report a higher response rate in HPV-positive than in HPV-negative patients (57). Immunological analyses found that an HPV-positive status contributes to T-cell infiltration and enhanced cytolytic activity which result in a better response to anti PD-1/PD-L1 therapy (57). Nevertheless, significant PD-L1 expression can also be observed in HPV-negative H&NCs and thus, the HPV status is not a prerequisite for immunotherapy in these tumors (62).

### HPV Integration Signatures and Viral DNA Used as Specific Tumor Markers

HPV DNA integration pattern can be used as specific tumor markers helpful for personalized patient follow-up (34, 63). This pattern encompasses several parameters, (I) locus at the molecular level, (II) break locus on the viral genome, (III) deletion of part of the viral genome, and (IV) characteristic patterns of the viral/cell genome junctions. The high specificity of this signature is essential for diagnostic purposes. For instance, in a patient previously treated for an HPV-associated tumor and developing a second tumor, the differential diagnosis between a metastasis versus the *de novo* development of an independent second tumor associated with the same viral genotype may be difficult. In this situation, the presence of the same insertional signature in the two lesions confers a very high level of specificity for a diagnosis of metastasis. As an example, we have demonstrated recently that a carcinoma developed on the base of the tongue in a male patient previously treated for a carcinoma of the anal canal shared the same HPV16 specific insertion molecular signature as that characterizing the anal tumor and we could conclude that the tongue tumor corresponded unambiguously to the metastasis of the primary anal tumor (64). The distinction can be important since the therapeutic approach is different in case of metastasis vs a second primary tumor. A limitation of this approach for diagnostic purpose relies on the fact that 30–40% of H&NCs harbor only episomal HPV DNA and thus HPV-chromosome insertional signatures are lacking. However, the identification of the precise lineage and sub-lineage of the HPV viral genotype should nevertheless provide a valuable tumor marker, particularly when different HPV strains/genotypes are detected in the respective heterogeneous tumors.

## Perspectives

Three major bio-clinical perspectives may be considered, (a) the development of the detection of circulating tumor DNA (ctDNA) using HPV sequences as a tumor marker, (b) the introduction of NGS methods for the optimal molecular characterization of viral DNA in clinical oncology, and (c) the analysis of the tumor immune microenvironment in the frame of immunotherapeutic protocols.

### HPV Genome: A Tumor Marker for the Detection of ctDNA

#### Circulating HPV DNA: A specific Form of ctDNA

Numerous applications of ctDNA as diagnostic and predictive/prognostic marker in oncology are under development (65–67). In most models, ctDNA is detected *via* the presence of somatic mutations, but the low rate of target molecules, especially in early stage tumors, implies that their detection can be affected by stochastic sampling leading to a lack of target molecules in some specimens (68), a limitation for clinical applications, (69). In this context, HPV-associated tumors represent a privileged model for the detection of ctDNA. As mentioned above, the HPV genome is a 7.8 kbp-long circular double-strand DNA molecule present in the nucleus of tumor cells, as free episomes and/or as an integrated form, in copy numbers varying from a few to thousands per cell. The viral DNA fragments shed in the blood corresponds thus to a large target of foreign DNA that can be more easily detected than ctDNA fragments harboring point mutations dispersed among germline circulating DNA. Indeed, circulating HPV DNA can be detected in patients with various types of HPV-associated carcinoma (70, 71). Studies found no circulating viral DNA in non-tumor patients or in patients with intra-epithelial neoplasia and circulating HPV DNA, referred to as ctHPV DNA, can thus be considered as a specific form of ctDNA (71) and serve as a tumor marker for improved diagnosis, prognosis, and treatment monitoring (72).

#### ctHPV DNA: A Diagnostic Marker

At the time of diagnosis of HPV-positive H&NCs, the rates of ctHPV DNA positivity ranged from 60.5 to 95.9% (57, 70, 71, 73, 74). In a study focused on early stage H&NCs and using an NGS-based approach for the detection of HPV16, all cases (55/55) were ctHPV DNA positive (75). Although a positive correlation between ctHPV DNA levels and tumor stages has been observed (71, 76, 77), a striking and recurrent fact is that small tumors may be associated with high ctHPV DNA levels whereas low levels are found in more advanced cases (70, 71, 74). The reasons for this discrepancy are unclear. It could be related to variations in tumor viral load and/or integration signatures, but the comparison between tumor viral loads and ctHPV DNA levels revealed only a weak correlation for H&NCs (70). Other factors such as tumor differentiation, necrosis, proliferative rate, or immune response might be involved in the release of viral DNA. Like in other tumor models, the data collected suggest that ctHPV DNA levels are not simply associated with tumor burden or the number of dying cells but they correspond to a complex combination of tumor biology factors, potentially playing an active role in immunomodulation or in other processes regulating cell homeostasis (68).

From these works, three major conclusions can be drawn. (I) High rates of ctHPV DNA positivity are observed at diagnosis in patients with HPV-associated H&NCs; (II) The ctHPV DNA level is poorly related to tumor volume; (III) ctHPV DNA is already detectable in patients with subclinical disease. In clinical oncology, the first potential application is the possibility of using ctHPV DNA detection as an alternative approach to histology for the diagnosis of HPV-associated invasive carcinoma. This new approach could notably be used for the diagnosis of relapse in patients previously treated for an HPV-associated tumor and presenting abnormal imaging. In this situation, the detection of ctHPV DNA should be a sufficient criterion to confirm the diagnosis of relapse, avoiding unnecessary biopsy procedure that may cause morbidity. However, the question of the specificity of this approach has to be considered since some cases may be difficult to interpret. We previously provided a proof of concept showing that the highly specific viral insertional signature could be detected in the blood of patients (78) and that this approach provides a valuable tool to ascertain the specificity of the result of ctHPV DNA analysis when necessary.

#### Dynamic of ctHPV DNA Load: A Prognostic/Predictive Marker

The comparison between virological and clinical data showed that ctHPV DNA load at diagnosis is poorly indicative of disease outcome (76, 77). This is in contrast with the Epstein-Barr virus (EBV)-associated nasopharyngeal carcinoma model in which patients with a high plasma EBV load at diagnosis present a higher tumor stage and metastatic status (79). This discrepancy may be related to a poor correlation between tumor size and HPV ctDNA load in H&NCs. In contrast, longitudinal studies report that the dynamics of ctHPV DNA load under treatment can be a surrogate for the quality of tumor response and represent a valuable prognostic factor (70, 75, 77). Response kinetics to chemoradiation showed a high degree of heterogeneity among patients, but a drop in ctHPV DNA load was observed in most cases, preceding tumor regression at imaging (70). In particular, the post-therapeutic ctHPV DNA load demonstrated a major surrogate marker for the quality of the tumor response (75). At the end of treatment, early relapse in patients with positive ctHPV DNA was observed whereas, in patients showing abnormal fixation at imaging and ctHPV DNA negative detection, no residual disease was detected (75). Altogether, these observations suggest that post treatment ctHPV DNA status has positive as well as negative predictive values.

The high level of sensitivity and specificity of ctHPV DNA as a tumor marker in patients with HPV-associated carcinomas advocates for use of this marker to improve the biological follow-up of patients. The clinical relevance of this approach has been documented in colon carcinomas (80). However, there is no current evidence that, in all H&NCs cases, a biological relapse characterized by ctHPV DNA positivity would be followed by a clinical relapse at short term. Prospective studies are necessary to document and provide the clinical validation of this approach. Other pending questions are to address the specificity of the test, as well as the clinical utility of this approach. Concerning the specificity, the criteria necessary to affirm a biological relapse should be determined according to the clinical situation. In case of doubt, the specificity of the insertional signature would be a formal argument for a diagnosis of relapse and discard the possibility of the subclinical development of a second HPV-associated tumor. The clinical utility of the detection of subclinical relapse has also to be discussed. What should be the attitude in case of ctHPV DNA positivity in patients with no clinical or radiological evidence of a tumor? The design of innovative therapeutic protocols targeting subclinical diseases will be necessary to address this question and, in this perspective, ctHPV DNA will be a valuable surrogate marker to measure the efficiency of these treatments.

### NGS for the Improved Characterization of HPV-Associated H&NCs

**Figure 1:**
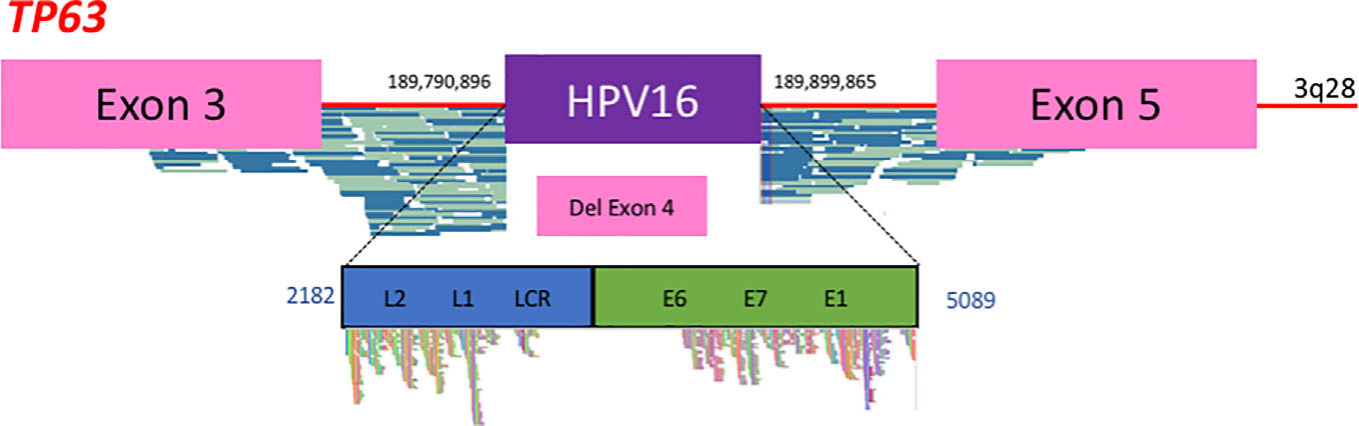
Next-generation sequencing data using the CaptHPV assay. The results presented here are sequences obtained from tumor tissue analyze in a patient with a HPV16-induced carcinoma. DNA sequences show viral DNA inserted within the *TP63* gene (3q28 chromosomal band), between exon 3 and exon 5 with gene disruption and loss of the exon 4. All genomics coordinates are presented in *Hg38* reference genome.

The detection of HPV DNA in biological specimens is commonly performed using q-PCR technique. However, this technology is not sufficient to allow the identification of the specific molecular markers useful for the diagnostic or follow-up purposes described above. In contrast, NGS is a powerful tool able to provide, in a single experiment, the extensive molecular characterization of HPV DNA in tumors: the complete nucleotide sequence of the viral genome (genotyping), its host chromosomal modifications (deletion, amplification), physical status, integration signature, chromosomal integration site(s), and identification of the gene(s) located in the vicinity (36, 81–84) (**Figure 1**). For instance, the CaptHPV method that we have developed (36) includes a double capture of HPV DNA fragments using single-stranded biotinylated probes recognizing 235 unique HPV types and variants. DNA sequences captured are sequenced using MiSeq instruments and raw sequencing data are aligned on the 235 HPV reference strains. A second alignment on human genome is performed to select hybrid reads that align on both HPV and human genomes and a map of the HPV pattern is deduced. This global approach has been validated technically and may be used in clinical oncology. In H&NCs, most of the data described above concerning integration targets were obtained using NGS methods (39–41, 75). These data have identified recurrent integration sites (39) and have shown that, as in genital tumors, viral insertion could impact the host genome by amplification of oncogenes and disruption of tumor suppressors as well as by driving inter- and intrachromosomal rearrangements (40). The clinical relevancy of the HPV integration status has been suggested (39) and, if confirmed, represents an important biological parameter to take into account when defining therapeutic strategies. Moreover, NGS approaches provide precise identifications of the viral sequences associated with the tumor, corresponding either to a specific HPV16 strain (75) or to any other genotypes (20% of the cases) (39, 40), genotypes that constitute valuable tumor markers.

Importantly, the NGS-based method developed for the molecular characterization of HPV DNA in cervical tumors can be successfully applied to the detection and characterization of ctHPV DNA (36). The NGS approach applied to the analysis of a standard blood sample allows the identification of any HPV genotype as well as the characterization of the insertional signature. Using this methodology, a diagnosis of HPV-associated invasive carcinoma can be obtained from a blood sample whatever the viral genotype involved. A prospective study is in progress to provide the clinical validation of this approach as an alternative to histology for a diagnosis of HPV-associated carcinoma, and to determine its limitation in terms of sensitivity and specificity (Sastre-Garau et al., in preparation). Furthermore, an NGS approach can be designed to combine the characterization of HPV DNA with the identification of somatic mutations frequent in HPV negative tumors. Using such a combined approach, Wang et al. detected ctDNA in 86 to 100% of HPV-positive or -negative H&NCs cases (57). Therefore, the design of NGS approaches combining extensive HPV DNA analysis and the detection of recurrent mutations can allow the determination of molecular markers and targets associated with in H&NCs independently of their HPV status (29). For the moment, NGS-based approaches remain relatively expensive and are not used routinely for sequential analyses during patient’ follow-up care in most hospitals or clinics worldwide. Their implementation requires specific facilities, including dedicated bioinformatics pipeline and trained team for technical processing and data interpretation. However, once we have a full picture of the HPV integration pattern that characterizes each tumor using NGS, the relevant markers determined can be extrapolated and sequentially analyzed using specific q-PCR method, allowing optimized long-term follow-up at reduced costs.

### Analysis of the Tumor Immune Microenvironment

The important role of the immune microenvironment as a major feature for tumor response to therapies of subsequent disease outcome has been underlined. Among the various parameters that can be analyzed, includes the density of the effector T cells (CD8+) and of PD-L1 in both immune cells and tumor cells that represent key prognostic and predictive parameters recurrently found in several studies (58–60). The development of immunotherapy protocols will require the evaluation of these markers using standardized parameters.

## Conclusion

Over the last 10 years, a number of viro-clinical studies have permitted us to obtain a better knowledge of the prevalence of HPV association in H&NCs, including the viral epidemiology and the oncogenesis of these tumors, allowing us to evolve from the concept of HPV-positive towards that of HPV-driven H&NCs (85). However, the use of HPV DNA as a diagnostic and/or predictive marker is not yet fully developed, in large part due to a lack of clinical validation. In the process of validation of tumor biomarkers, three main steps should be distinguished: analytical validity, clinical validity, and clinical utility (86). In the model of HPV-associated H&NCs, two major steps have already been reached: (I) the analytical validity of the HPV status using different methods (immunohistochemistry, PCR, or NGS) and (II) the clinical validity of the prognostic value of the HPV status when considered as a binary parameter (positive *versus* negative) (45). However, the clinical validity of other potential prognostic viral-related markers, such as the exact genotype or the physical state of viral DNA, needs to be further documented.

HPV-associated tumors represent a privileged model for the analysis of ctDNA and viral sequences constitute a very convenient biological role model to assess the course of the disease. ctHPV DNA is a sensitive tumor marker and this should facilitate the clinical validation of the “liquid biopsy approach” for the diagnosis of invasive carcinomas, for instance in case of suspicion of relapse. This validation will require the analysis of various tumors differing in size, localization, and viral genotype before the ctHPV DNA approach could be implemented as a recognized diagnostic tool in clinical oncology, avoiding more invasive procedures such as biopsies or fine needle aspirations. The ctDNA load at diagnosis is a poor prognostic marker, but its dynamic during the treatment is a promising predictive surrogate of tumor response. During the follow-up procedures, the clinical validation of ctHPV DNA as a predictive marker of relapse will solicit prospective studies demonstrating that all of the biological relapses precede clinical relapses. Difficulties need to be overcome. For instance, current studies report that advanced cases of HPV-positive tumors remain ctHPV DNA negative and the identification of the parameters accounting for this discrepancy is a prerequisite for a large use of ctDNA as a surrogate marker of the course of H&NCS.

Once these clinical validations are obtained, a major challenge remains to document the clinical utility of the diagnosis of sub-clinical disease, which depends mainly on the possibility of treatment. New tools will be necessary for the care of subclinical relapses and viral-associated tumors represent an attractive model for the development of immunotherapy. As an example, the treatment of high grade cervical intra-epithelial neoplasia using therapeutic HPV16/18 vaccine is currently in evaluation and provides encouraging results (87). Such an approach might be extended to sub-clinical diseases. The design of these innovative treatments as well as the assessment of their efficiency will require extensive molecular characterization of the viral sequences, and tools allowing this characterization are available.

The analysis of a large series of H&NCs using NGS approaches should enhance our knowledge about the biology of these tumors and favor further developments in diagnosis, follow-up, and treatments. The implementation of large data bases collecting the biological and clinical data obtained will be a powerful common tool favoring these advances.

## Author Contributions

XS-G wrote the manuscript and AH reviewed the manuscript. All authors contributed to the article and approved the submitted version.

## Conflict of Interest

The authors declare that the research was conducted in the absence of any commercial or financial relationships that could be construed as a potential conflict of interest.
